# Supplementary material for: Trends in Animal Shelter Management, Adoption, and Animal Death in Taiwan from 2012 to 2020
Source: Animals (Basel). 2023 Apr 24;13(9):1451. doi: 10.3390/ani13091451 (PMC10177604; doi:10.3390/ani13091451)
Supplement: Supplementary file 1 [file animals-13-01451-s001.zip › Table S2.pdf]

Table S2. The three-year average number and percentage (the number of the county over the total number in Taiwan) of public animal shelter intakes for each county in Taiwan from 2012 to 2020.

**County**	**2012 - 2014**	**2015 - 2017**	**2018 - 2020**
Chiayi County	3,971 (3.74%)	2,108 (3.38%)	684 (1.53%)
Chiayi City	1,137 (1.07%)	827 (1.33%)	313 (0.70%)
Changhua County	5,521 (5.20%)	2,377 (3.81%)	1,491 (3.32%)
Hsinchu County	2,870 (2.70%)	1,419 (2.28%)	508 (1.13%)
Hsinchu City	1,348 (1.27%)	878 (1.41%)	553 (1.23%)
Hualien County	2,941 (2.77%)	1,084 (1.74%)	513 (1.14%)
Kaohsiung	9,182 (8.64%)	5,152 (8.26%)	4,707 (10.49%)
Keelung County	1,871 (1.76%)	1,150 (1.85%)	433 (0.96%)
Kinmen County	1,244 (1.17%)	1,070 (1.72%)	884 (1.97%)
Lienchiang County	96 (0.09%)	18 (0.03%)	21 (0.05%)
Miaoli County	3,347 (3.15%)	1,698 (2.72%)	1,466 (3.27%)
Nantou County	5,558 (5.23%)	2,770 (4.44%)	539 (1.20%)
New Taipei	13,564 (12.76%)	8,907 (14.29%)	6,040 (13.46%)
Penghu County	1,098 (1.03%)	729 (1.17%)	538 (1.20%)
Pingtung County	5,477 (5.15%)	2,665 (4.28%)	731 (1.63%)
Taichung	9,496 (8.94%)	5,544 (8.90%)	7,656 (17.06%)
Tainan	11,472 (10.80%)	8,198 (13.15%)	7,595 (16.93%)
Taipei	6,023 (5.67%)	3,604 (5.78%)	2,757 (6.14%)
Taitung County	2,687 (2.53%)	1,414 (2.27%)	556 (1.24%)
Taoyuan	9,318 (8.77%)	6,120 (9.82%)	4,794 (10.68%)
Yilan County	3,963 (3.73%)	2,321 (3.72%)	1,508 (3.36%)
Yunlin County	4,085 (3.84%)	2,270 (3.64%)	584 (1.30%)
Average total[1]	106,270	62,322	44,873

[1]: the three-year average number of total number of public animal shelter intakes in Taiwan
