# Supplementary material for: Trends in Animal Shelter Management, Adoption, and Animal Death in Taiwan from 2012 to 2020
Source: Animals (Basel). 2023 Apr 24;13(9):1451. doi: 10.3390/ani13091451 (PMC10177604; doi:10.3390/ani13091451)
Supplement: Supplementary file 1 [file animals-13-01451-s001.zip › Table S3.pdf]

Table S3. The three-year average number and percentage (the number of the county over the total number in Taiwan) of public animal shelter outcomes for each county in Taiwan from 2012 to 2020.

**County**	**2012 - 2014**	**2015 - 2017**	**2018 - 2020**
Chiayi County	3,276 (3.62%)	2,068 (3.49%)	231 (0.74%)
Chiayi City	1,042 (1.15%)	819 (1.38%)	285 (0.92%)
Changhua County	4,082 (4.51%)	2,372 (4.01%)	1,173 (3.77%)
Hsinchu County	2,749 (3.04%)	1,418 (2.40%)	414 (1.33%)
Hsinchu City	1,237 (1.37%)	860 (1.45%)	343 (1.10%)
Hualien County	2,451 (2.71%)	1,061 (1.79%)	283 (0.91%)
Kaohsiung	6,107 (6.75%)	4,197 (7.09%)	4,245 (13.65%)
Keelung County	1,536 (1.70%)	1,144 (1.93%)	423 (1.36%)
Kinmen County	833 (0.92%)	675 (1.14%)	294 (0.95%)
Lienchiang County	81 (0.09%)	9 (0.02%)	18 (0.06%)
Miaoli County	2,783 (3.08%)	1,631 (2.76%)	652 (2.10%)
Nantou County	4,793 (5.30%)	2,724 (4.60%)	340 (1.09%)
New Taipei	10,491 (11.60%)	8,794 (14.85%)	5,922 (19.04%)
Penghu County	603 (0.67%)	693 (1.17%)	423 (1.36%)
Pingtung County	5,454 (6.03%)	2,711 (4.58%)	390 (1.25%)
Taichung	8,664 (9.58%)	4,933 (8.33%)	4,445 (14.29%)
Tainan	10,697 (11.83%)	8,251 (13.94%)	4,184 (13.45%)
Taipei	4,782 (5.29%)	3,489 (5.89%)	2,450 (7.88%)
Taitung County	2,404 (2.66%)	1,422 (2.40%)	547 (1.76%)
Taoyuan	8,896 (9.84%)	5,915 (9.99%)	2,553 (8.21%)
Yilan County	3,527 (3.90%)	1,793 (3.03%)	945 (3.04%)
Yunlin County	3,971 (4.39%)	2,222 (3.75%)	538 (1.73%)
Average total[1]	90,456	59,201	31,098

[1]: the three-year average number of total number of public animal shelter outcomes in Taiwan
