# Supplementary material for: Trends in Animal Shelter Management, Adoption, and Animal Death in Taiwan from 2012 to 2020
Source: Animals (Basel). 2023 Apr 24;13(9):1451. doi: 10.3390/ani13091451 (PMC10177604; doi:10.3390/ani13091451)
Supplement: Supplementary file 1 [file animals-13-01451-s001.zip › Table S4.pdf]

Table S4 Table 1. The complete table of total number and percentage of public animal shelter intakes for different reasons for each county from 2018 to 2020.

County	Capture	Consignation	Relinquishment	Rescue	Confiscation	Capture and Rescue	Other	Total
Chiayi County	1,830 (89.1%)	42 (2.0%)	10 (0.5%)	25 (1.2%)	16 (0.8%)	1,855 (90.4%)	130 (6.3%)	2,053
Chiayi City	391 (41.7%)	327 (34.9%)	147 (15.7%)	0 (0.0%)	11 (1.2%)	391 (41.7%)	62 (6.6%)	938
Changhua County	2,891 (64.6%)	985 (22.0%)	132 (3.0%)	340 (7.6%)	36 (0.8%)	3,231 (72.2%)	89 (2.0%)	4,473
Hsinchu County	361 (23.6%)	603 (39.5%)	108 (7.1%)	385 (25.2%)	69 (4.5%)	746 (48.8%)	2 (0.1%)	1,528
Hsinchu City	987 (59.4%)	536 (32.3%)	57 (3.4%)	67 (4.0%)	0 (0.0%)	1,054 (63.4%)	15 (0.9%)	1,662
Hualien County	946 (61.4%)	273 (17.7%)	7 (0.5%)	47 (3.1%)	4 (0.3%)	993 (64.5%)	263 (17.1%)	1,540
Kaohsiung	10,304 (72.9%)	1,458 (10.3%)	242 (1.7%)	1,834 (13.0%)	59 (0.4%)	12,138 (85.9%)	228 (1.6%)	14,125
Keelung County	598 (46.0%)	504 (38.8%)	15 (1.2%)	126 (9.7%)	45 (3.5%)	724 (55.7%)	11 (0.8%)	1,299
Kinmen County	2,155 (81.2%)	415 (15.6%)	33 (1.2%)	43 (1.6%)	0 (0.0%)	2,198 (82.8%)	7 (0.3%)	2,653
Lienchiang County	58 (90.6%)	0 (0.0%)	3 (4.7%)	3 (4.7%)	0 (0.0%)	61 (95.3%)	0 (0.0%)	64
Miaoli County	3,102 (70.1%)	1,032 (23.3%)	66 (1.5%)	206 (4.7%)	0 (0.0%)	3,308 (74.7%)	20 (0.5%)	4,426
Nantou County	1,041 (64.3%)	328 (20.3%)	95 (5.9%)	140 (8.7%)	4 (0.2%)	1,181 (73.0%)	10 (0.6%)	1,618
New Taipei	9,965 (55.0%)	5,807 (32.0%)	399 (2.2%)	990 (5.5%)	3 (0.0%)	10,955 (60.4%)	968 (5.3%)	18,132
Penghu County	1,120 (69.3%)	400 (24.8%)	30 (1.9%)	32 (2.0%)	0 (0.0%)	1,152 (71.3%)	34 (2.1%)	1,616
Pingtung County	1,073 (48.9%)	101 (4.6%)	7 (0.3%)	813 (37.1%)	6 (0.3%)	1,886 (86.0%)	193 (8.8%)	2,193
Taichung	10,652 (46.4%)	8,788 (38.3%)	877 (3.8%)	2,533 (11.0%)	118 (0.5%)	13,185 (57.4%)	1 (0.0%)	22,969
Tainan	13,425 (58.9%)	8,940 (39.2%)	402 (1.8%)	22 (0.1%)	0 (0.0%)	13,447 (59.0%)	0 (0.0%)	22,789
Taipei	546 (6.4%)	871 (10.2%)	355 (4.1%)	6,741 (78.6%)	0 (0.0%)	7,287 (85.0%)	63 (0.7%)	8,576
Taitung County	200 (12.0%)	1,342 (80.5%)	78 (4.7%)	30 (1.8%)	0 (0.0%)	230 (13.8%)	17 (1.0%)	1,667
Taoyuan	9,520 (66.2%)	1,929 (13.4%)	440 (3.1%)	2,175 (15.1%)	1 (0.0%)	11,695 (81.3%)	317 (2.2%)	14,382
Yilan County	2,078 (45.9%)	659 (14.6%)	469 (10.4%)	1,318 (29.1%)	0 (0.0%)	3,396 (75.1%)	0 (0.0%)	4,524
Yunlin County	1,340 (76.4%)	0 (0.0%)	16 (0.9%)	116 (6.6%)	267 (15.2%)	1,456 (83.1%)	14 (0.8%)	1,753

Table S4 Table 2. The complete table of total number and percentage of public animal shelter outcomes for different reasons for each county from 2018 to 2020.

County	Reclamation	Adoption	Euthanasia	Unassisted death	TNVR[1]	Escape	Other	Total
Chiayi County	28 (1.3%)	538 (25.5%)	6 (0.3%)	120 (5.7%)	1,324 (62.8%)	27 (1.3%)	64 (3.0%)	2,107
Chiayi City	55 (6.0%)	740 (80.2%)	24 (2.6%)	35 (3.8%)	60 (6.5%)	8 (0.9%)	1 (0.1%)	923
Changhua County	136 (3.1%)	2,876 (65.1%)	0 (0.0%)	508 (11.5%)	819 (18.6%)	76 (1.7%)	0 (0.0%)	4,415
Hsinchu County	78 (5.5%)	1,033 (72.3%)	0 (0.0%)	135 (9.4%)	143 (10.0%)	39 (2.7%)	1 (0.1%)	1,429
Hsinchu City	103 (6.5%)	713 (45.0%)	32 (2.0%)	182 (11.5%)	525 (33.2%)	26 (1.6%)	2 (0.1%)	1,583
Hualien County	59 (3.7%)	692 (43.7%)	42 (2.7%)	56 (3.5%)	731 (46.1%)	4 (0.3%)	0 (0.0%)	1,584
Kaohsiung	658 (4.8%)	11,333 (83.0%)	0 (0.0%)	746 (5.5%)	235 (1.7%)	37 (0.3%)	646 (4.7%)	13,655
Keelung County	216 (16.1%)	902 (67.2%)	0 (0.0%)	150 (11.2%)	1 (0.1%)	14 (1.0%)	59 (4.4%)	1,342
Kinmen County	133 (5.1%)	661 (25.3%)	6 (0.2%)	82 (3.1%)	1,726 (66.1%)	0 (0.0%)	2 (0.1%)	2,610
Lienchiang County	11 (20.8%)	38 (71.7%)	0 (0.0%)	4 (7.5%)	0 (0.0%)	0 (0.0%)	0 (0.0%)	53
Miaoli County	117 (2.7%)	1,189 (27.4%)	35 (0.8%)	625 (14.4%)	2,254 (52.0%)	112 (2.6%)	3 (0.1%)	4,335
Nantou County	159 (9.6%)	758 (45.7%)	19 (1.1%)	84 (5.1%)	632 (38.1%)	5 (0.3%)	0 (0.0%)	1,657
New Taipei	2,193 (12.2%)	14,657 (81.4%)	0 (0.0%)	913 (5.1%)	47 (0.3%)	62 (0.3%)	129 (0.7%)	18,001
Penghu County	51 (3.8%)	716 (52.8%)	38 (2.8%)	530 (39.1%)	0 (0.0%)	14 (1.0%)	8 (0.6%)	1,357
Pingtung County	48 (2.3%)	1,088 (51.1%)	11 (0.5%)	22 (1.0%)	959 (45.0%)	0 (0.0%)	2 (0.1%)	2,130
Taichung	1,183 (5.1%)	10,570 (45.9%)	97 (0.4%)	1,485 (6.4%)	9,603 (41.7%)	110 (0.5%)	0 (0.0%)	23,048
Tainan	777 (3.5%)	10,748 (48.4%)	0 (0.0%)	1,026 (4.6%)	9,595 (43.2%)	52 (0.2%)	2 (0.0%)	22,200
Taipei	2,029 (25.0%)	5,364 (66.1%)	9 (0.1%)	554 (6.8%)	3 (0.0%)	141 (1.7%)	17 (0.2%)	8,117
Taitung County	122 (7.4%)	1,488 (90.1%)	10 (0.6%)	21 (1.3%)	2 (0.1%)	8 (0.5%)	1 (0.1%)	1,652
Taoyuan	607 (4.3%)	6,481 (46.3%)	50 (0.4%)	525 (3.7%)	5,032 (35.9%)	68 (0.5%)	1,245 (8.9%)	14,008
Yilan County	374 (8.6%)	2,054 (47.1%)	38 (0.9%)	370 (8.5%)	0 (0.0%)	0 (0.0%)	1,521 (34.9%)	4,357
Yunlin County	2 (0.1%)	1,298 (79.2%)	0 (0.0%)	313 (19.1%)	0 (0.0%)	5 (0.3%)	20 (1.2%)	1,638

[1]: trap-neuter-vaccinate-return
