# Supplementary material for: Trends in Animal Shelter Management, Adoption, and Animal Death in Taiwan from 2012 to 2020
Source: Animals (Basel). 2023 Apr 24;13(9):1451. doi: 10.3390/ani13091451 (PMC10177604; doi:10.3390/ani13091451)
Supplement: Supplementary file 1 [file animals-13-01451-s001.zip › Table S5.pdf]

Table S5. Univariable linear regression results

Table S5 Table 1. Univariable linear regression results for the number of shelter animal intakes per month over the maximum shelter capacity from 2012 to 2020.

**Covariate**	**Category**	**Estimate (95% CI[1])**	***P*-value**	**P-value for the covariate**
Year	Intercept	146.56 (127.02 to 166.09)	<0.001	<0.001
	Year	-15.70 (-19.81 to -11.60)	<0.001	
County	Intercept (Taipei)	56.38 (12.71 to 100.05)	0.012	<0.001
	Chiayi County	114.41 (52.65 to 176.17)	<0.001	
	Chiayi City	-3.69 (-65.45 to 58.07)	0.907	
	Changhua County	47.53 (-14.23 to 109.30)	0.133	
	Hsinchu County	49.37 (-12.40 to 111.13)	0.119	
	Hsinchu City	-37.07 (-98.83 to 24.70)	0.241	
	Hualien County	69.70 (7.94 to 131.46)	0.028	
	Kaohsiung	-0.71 (-62.47 to 61.05)	0.982	
	Keelung County	7.58 (-54.18 to 69.34)	0.810	
	Kinmen and Lienchiang County	-32.01 (-93.77 to 29.75)	0.311	
	Miaoli County	3.90 (-57.86 to 65.66)	0.902	
	Nantou County	10.20 (-51.56 to 71.96)	0.747	
	New Taipei	-16.68 (-78.44 to 45.08)	0.597	
	Penghu County	-40.66 (-102.42 to 21.11)	0.199	
	Pingtung County	211.52 (149.76 to 273.28)	<0.001	

	Taichung	69.70 (7.94 to 131.46)	0.028	
	Tainan	43.29 (-18.47 to 105.05)	0.171	
	Taitung County	51.43 (-10.33 to 113.20)	0.105	
	Taoyuan	9.74 (-52.02 to 71.51)	0.758	
	Yilan County	9.20 (-52.56 to 70.96)	0.771	
	Yunlin County	7.90 (-53.86 to 69.66)	0.802	
Administrative division[2]	Intercept (County)	113.89 (97.66 to 130.13)	<0.001	<0.001
	City	-68.58 (-102.38 to -34.77)	<0.001	
	Metropolis	-39.96 (-66.48 to -13.44)	0.004	
	Outer island	-93.85 (-133.63 to -54.07)	<0.001	
Geographical division[3]	Intercept (West)	84.22 (61.43 to 107.02)	<0.001	<0.001
	East	32.72 (-9.92 to 75.36)	0.134	
	North	-24.68 (-54.52 to 5.16)	0.107	
	Outer island	-64.18 (-106.82 to -21.54)	0.004	
	South	45.12 (12.89 to 77.35)	0.007	
Per capita gross domestic product (GDP)	Intercept	545.72 (423.40 to 668.04)	<0.001	<0.001
	Every 1000 New Taiwan Dollar	-0.63 (-0.79 to -0.46)	<0.001	
Population	Intercept	86.51 (69.21 to 103.81)	<0.001	0.662
	Every 100,000 people	-0.25 (-1.35 to 0.86)	0.662	
Higher education[4]	Intercept	172.33 (132.46 to 212.20)	<0.001	<0.001
	%	-2.31 (-3.30 to -1.31)	<0.001	
Plain area of a county	Intercept	49.51 (31.91 to 67.11)	<0.001	<0.001

	10 km^2	0.74 (0.45 to 1.03)	<0.001	
Population density over the plain area	Intercept	90.60 (76.88 to 104.33)	<0.001	0.048
	100,000 people / 1 km^2	-1.14 (-2.25E to -0.02)	0.048	
Population density over the county area	Intercept	96.23 (81.73 to 110.72)	<0.001	0.004
	Person / 1 km^2	-0.01 (-0.01 to -2.59E-03)	0.004	
Fertility rate	Intercept	144.94 (81.29 to 208.60)	<0.001	0.057
	‰	-1.90 (-3.84 to 0.04)	0.057	
Euthanasia[5]	Intercept (No)	40.55 (24.44 to 56.66)	<0.001	<0.001
	Yes	77.75 (56.14 to 99.36)	<0.001	

[1]: confidence interval

[2]: administrative divisions in Taiwan (i.e., metropolis, city, county, and outer island)

[3]: geographical divisions (i.e., north, south, west, east, and outer island)

[4]: percentage of people above 15 years old with a bachelor's degree by county and month

[5]: whether euthanasia for population control was enforced

Table S5 Table 2. Univariable linear regression results for the number of shelter animal outcomes per month over the maximum shelter capacity from 2012 to 2020.

Covariate	Category	Estimate (95% CI[1])	*P*-value	P-value for the covariate
Year	Intercept	133.32 (114.43 to 152.21)	<0.001	<0.001
	Year	-15.12 (-19.09 to -11.15)	<0.001	
County	Intercept (Taipei)	48.84 (5.83 to 91.86)	0.027	<0.001
	Chiayi County	91.92 (31.08 to 152.76)	0.004	
	Chiayi City	0.82 (-60.02 to 61.66)	0.979	
	Changhua County	35.56 (-25.28 to 96.39)	0.254	
	Hsinchu County	52.14 (-8.69 to 112.98)	0.095	
	Hsinchu City	-31.90 (-92.74 to 28.94)	0.306	
	Hualien County	56.57 (-4.27 to 117.40)	0.070	
	Kaohsiung	-6.30 (-67.14 to 54.54)	0.839	
	Keelung County	8.62 (-52.22 to 69.46)	0.782	
	Kinmen and Lienchiang County	-34.89 (-95.72 to 25.95)	0.263	
	Miaoli County	-1.92 (-62.76 to 58.92)	0.951	
	Nantou County	10.16 (-50.68 to 70.99)	0.744	
	New Taipei	-13.73 (-74.57 to 47.10)	0.659	
	Penghu County	-37.41 (-98.25 to 23.43)	0.230	
	Pingtung County	209.47 (148.63 to 270.30)	<0.001	
	Taichung	51.39 (-9.45 to 112.23)	0.100	
	Tainan	35.69 (-25.15 to 96.53)	0.252	

	Taitung County	52.38 (-8.46 to 113.22)	0.093	
	Taoyuan	7.90 (-52.94 to 68.74)	0.799	
	Yilan County	3.89 (-56.95 to 64.73)	0.900	
	Yunlin County	13.48 (-47.36 to 74.32)	0.665	
Administrative division[2]	Intercept (County)	101.21 (85.40 to 117.01)	<0.001	<0.001
	City	-59.85 (-92.74 to -26.95)	<0.001	
	Metropolis	-39.87 (-65.68 to -14.07)	0.003	
	Outer island	-88.51 (-127.22 to -49.80)	<0.001	
Geographical division[3]	Intercept (West)	70.58 (48.34 to 92.81)	<0.001	<0.001
	East	32.74 (-8.86 to 74.34)	0.125	
	North	-17.89 (-47.00 to 11.23)	0.230	
	Outer island	-57.88 (-99.48 to -16.28)	0.007	
	South	44.59 (13.14 to 76.03)	0.006	
Per capita gross domestic product (GDP)	Intercept	508.30 (389.26 to 627.34)	<0.001	<0.001
	New Taiwan Dollar	-5.91E-04 (-7.51E-04 to -4.30E-04)	<0.001	
Population	Intercept	76.68 (59.97 to 93.39)	<0.001	0.530
	Number of people	-3.43E-06 (-1.41E-05 to 7.26E-06)	0.530	
Higher education[4]	Intercept	154.29 (115.56 to 193.01)	<0.001	<0.001
	%	-2.12 (-3.09 to -1.16)	<0.001	
Plain area of a county	Intercept	42.47 (25.29 to 59.66)	<0.001	<0.001
	10,000 m^2	6.57E-04 (3.73E-04 to 9.41E-04)	<0.001	
Population density over the plain area	Intercept	78.65 (65.36 to 91.95)	<0.001	0.082

	Person / 10,000 m^2	-9.63E-04 (-2.04E-03 to 1.18E-04)	0.082	
Population density over the county area	Intercept	83.75 (69.69 to 97.82)	<0.001	0.009
	Person / 1 km^2	-6.91E-03 (-1.21E-02 to -1.75E-03)	0.009	
Fertility rate	Intercept	124.88 (63.23 to 186.53)	<0.001	0.094
	‰	-1.61 (-3.49 to 0.26)	0.094	
Euthanasia[5]	Intercept (No)	29.49 (14.09 to 44.89)	<0.001	<0.001
	Yes	78.02 (57.35 to 98.68)	<0.001	

[1]: confidence interval

[2]: administrative divisions in Taiwan (i.e., metropolis, city, county, and outer island)

[3]: geographical divisions (i.e., north, south, west, east, and outer island)

[4]: percentage of people above 15 years old with a bachelor's degree by county and month

[5]: whether euthanasia for population control was enforced

Table S5 Table 3. Univariable linear regression results for the number of adopted animals over the shelter animal intakes per month from 2012 to 2020.

**Covariate**	**Category**	**Estimate (95% CI[1])**	***P*-value**	**P-value for the covariate**
Year	Intercept	41.28 (34.92 to 47.64)	<0.001	<0.001
	Year	4.13 (2.80 to 5.47)	<0.001	
County	Intercept (Taipei)	80.35 (65.70 to 94.99)	<0.001	<0.001
	Chiayi County	-26.34 (-47.05 to -5.62)	0.014	
	Chiayi City	2.94 (-17.78 to 23.65)	0.782	
	Changhua County	-30.34 (-51.06 to -9.63)	0.005	
	Hsinchu County	-13.50 (-34.21 to 7.22)	0.203	
	Hsinchu City	-18.31 (-39.03 to 2.41)	0.085	
	Hualien County	-26.87 (-47.58 to -6.15)	0.012	
	Kaohsiung	-13.93 (-34.65 to 6.78)	0.189	
	Keelung County	-17.73 (-38.44 to 2.99)	0.095	
	Kinmen and Lienchiang County	-56.52 (-77.24 to -35.81)	<0.001	
	Miaoli County	-40.51 (-61.22 to -19.79)	<0.001	
	Nantou County	-39.82 (-60.54 to -19.11)	<0.001	
	New Taipei	-1.52 (-22.23 to 19.20)	0.886	
	Penghu County	-37.46 (-58.17 to -16.74)	<0.001	
	Pingtung County	-41.84 (-62.56 to -21.13)	<0.001	
	Taichung	-25.22 (-45.94 to -4.51)	0.018	
	Tainan	-13.55 (-34.26 to 7.17)	0.202	

	Taitung County	-1.82 (-22.53 to 18.90)	0.864	
	Taoyuan	-15.40 (-36.12 to 5.31)	0.147	
	Yilan County	-39.80 (-60.52 to -19.09)	<0.001	
	Yunlin County	-15.66 (-36.37 to 5.06)	0.140	
Administrative division[2]	Intercept (County)	52.70 (47.71 to 57.69)	<0.001	<0.001
	City	16.62 (6.23 to 27.00)	0.002	
	Metropolis	16.05 (7.90 to 24.19)	<0.001	
	Outer island	-19.34 (-31.56 to -7.12)	0.002	
Geographical division[3]	Intercept (West)	50.04 (42.82 to 57.25)	<0.001	<0.001
	East	15.97 (2.47 to 29.47)	0.022	
	North	15.13 (5.68 to 24.58)	0.002	
	Outer island	-16.68 (-30.18 to -3.18)	0.016	
	South	11.77 (1.56 to 21.97)	0.025	
Per capita gross domestic product (GDP)	Intercept	-73.58 (-112.74 to -34.42)	<0.001	<0.001
	New Taiwan Dollar	1.78E-04 (1.25E-04 to 2.31E-04)	<0.001	
Population	Intercept	50.89 (45.66 to 56.11)	<0.001	<0.001
	Number of people	6.19E-06 (2.85E-06 to 9.54E-06)	<0.001	
Higher education[4]	Intercept	21.51 (9.57 to 33.46)	<0.001	<0.001
	%	0.95 (0.65 to 1.24)	<0.001	
Plain area of a county	Intercept	58.84 (52.99 to 64.69)	<0.001	0.652
	10,000 m^2	-2.23E-05 (-1.19E-04 to 7.44E-05)	0.652	
Population density over the plain area	Intercept	54.99 (50.74 to 59.24)	<0.001	0.009

	Person / 10,000 m^2	4.67E-04 (1.21E-04 to 8.13E-04)	0.009	
Population density over the county area	Intercept	52.11 (47.70 to 56.52)	<0.001	<0.001
	Person / 1 km^2	3.61E-03 (2.00E-03 to 5.23E-03)	<0.001	
Fertility rate	Intercept	76.51 (56.62 to 96.40)	<0.001	0.062
	‰	-0.58 (-1.19 to 0.03)	0.062	
Euthanasia[5]	Intercept (No)	67.53 (62.19 to 72.87)	<0.001	<0.001
	Yes	-17.49 (-24.66 to -10.32)	<0.001	

[1]: confidence interval

[2]: administrative divisions in Taiwan (i.e., metropolis, city, county, and outer island)

[3]: geographical divisions (i.e., north, south, west, east, and outer island)

[4]: percentage of people above 15 years old with a bachelor's degree by county and month

[5]: whether euthanasia for population control was enforced

Table S5 Table 4. Univariable linear regression results for the number of unassisted death animals over the shelter animal intakes per year from 2012 to 2020.

Covariate	Category	Estimate (95% CI[1])	*P*-value	P-value for the covariate
Year	Intercept	11.83 (9.39 to 14.27)	<0.001	0.072
	Year	-0.63 (-1.30 to 0.05)	0.072	
County	Intercept (Taipei)	7.02 (1.76 to 12.28)	0.010	<0.001
	Chiayi County	10.38 (2.95 to 17.82)	0.007	
	Chiayi City	-2.44 (-9.88 to 5.00)	0.521	
	Changhua County	6.63 (-0.81 to 14.06)	0.083	
	Hsinchu County	-0.16 (-7.59 to 7.28)	0.967	
	Hsinchu City	4.29 (-3.15 to 11.72)	0.261	
	Hualien County	-0.98 (-8.41 to 6.46)	0.797	
	Kaohsiung	6.69 (-0.75 to 14.13)	0.080	
	Keelung County	3.15 (-4.29 to 10.58)	0.409	
	Kinmen and Lienchiang County	-1.52 (-8.96 to 5.92)	0.689	
	Miaoli County	11.45 (4.01 to 18.88)	0.003	
	Nantou County	7.54 (0.10 to 14.98)	0.049	
	New Taipei	1.02 (-6.42 to 8.46)	0.789	
	Penghu County	16.12 (8.68 to 23.56)	<0.001	
	Pingtung County	-3.50 (-10.93 to 3.94)	0.358	
	Taichung	0.04 (-7.40 to 7.47)	0.992	
	Tainan	-0.14 (-7.57 to 7.30)	0.971	

	Taitung County	-4.14 (-11.58 to 3.30)	0.277	
	Taoyuan	-2.41 (-9.85 to 5.02)	0.526	
	Yilan County	4.11 (-3.33 to 11.55)	0.281	
	Yunlin County	5.40 (-2.03 to 12.84)	0.157	
Administrative division[2]	Intercept (County)	10.70 (8.75 to 12.65)	<0.001	0.062
	City	-2.01 (-6.07 to 2.05)	0.334	
	Metropolis	-2.81 (-5.99 to 0.38)	0.086	
	Outer island	3.63 (-1.15 to 8.40)	0.139	
Geographical division[3]	Intercept (West)	13.23 (10.56 to 15.91)	<0.001	0.002
	East	-8.77 (-13.77 to -3.77)	<0.001	
	North	-4.78 (-8.28 to -1.28)	0.008	
	Outer island	1.09 (-3.91 to 6.09)	0.670	
	South	-4.01 (-7.79 to -0.23)	0.039	
Higher education[4]	Intercept	14.82 (10.02 to 19.61)	<0.001	0.040
	%	-0.12 (-0.24 to -0.01)	0.040	
Euthanasia[5]	Intercept (No)	8.86 (7.07 to 10.65)	<0.001	0.071
	Yes	2.55 (-0.19 to 5.29)	0.071	

[1]: confidence interval

[2]: administrative divisions in Taiwan (i.e., metropolis, city, county, and outer island)

[3]: geographical divisions (i.e., north, south, west, east, and outer island)

[4]: percentage of people above 15 years old with a bachelor's degree by county and month

[5]: whether euthanasia for population control was enforced

Table S5 Table 5. Univariable linear regression results for the shelter animal intakes per month over the maximum shelter capacity from 2018 to 2020.

Covariate	Category	Estimate (95% CI[1])	*P*-value	P-value for the covariate
Year	Intercept	32.98 (29.19 to 36.78)	<0.001	<0.001
	Year	7.09 (4.15 to 10.03)	<0.001	
Month	Intercept	33.77 (28.60 to 38.93)	<0.001	0.007
	Month	0.97 (0.27 to 1.67)	0.007	
County	Intercept (Taipei)	39.05 (31.95 to 46.16)	<0.001	<0.001
	Chiayi County	12.79 (2.75 to 22.84)	0.013	
	Chiayi City	-17.33 (-27.38 to -7.28)	<0.001	
	Changhua County	10.45 (0.40 to 20.50)	0.042	
	Hsinchu County	-5.37 (-15.42 to 4.68)	0.295	
	Hsinchu City	-27.50 (-37.55 to -17.45)	<0.001	
	Hualien County	3.73 (-6.32 to 13.77)	0.468	
	Kaohsiung	2.25 (-7.80 to 12.30)	0.660	
	Keelung County	-15.00 (-25.05 to -4.95)	0.004	
	Kinmen and Lienchiang County	-19.19 (-29.24 to -9.14)	<0.001	
	Miaoli County	1.93 (-8.12 to 11.98)	0.707	
	Nantou County	-26.92 (-36.97 to -16.87)	<0.001	
	New Taipei	-13.81 (-23.85 to -3.76)	0.007	
	Penghu County	-28.31 (-38.36 to -18.26)	<0.001	
	Pingtung County	27.16 (17.12 to 37.21)	<0.001	

	Taichung	88.55 (78.50 to 98.60)	<0.001	
	Tainan	44.24 (34.19 to 54.29)	<0.001	
	Taitung County	-0.46 (-10.51 to 9.58)	0.928	
	Taoyuan	7.94 (-2.10 to 17.99)	0.122	
	Yilan County	-0.97 (-11.02 to 9.07)	0.849	
	Yunlin County	-22.82 (-32.87 to -12.77)	<0.001	
Administrative division[2]	Intercept (County)	39.00 (35.86 to 42.15)	<0.001	<0.001
	City	-19.90 (-26.44 to -13.35)	<0.001	
	Metropolis	21.58 (16.44 to 26.72)	<0.001	
	Outer island	-23.70 (-31.41 to -16.00)	<0.001	
Geographical division[3]	Intercept (West)	49.29 (44.61 to 53.97)	<0.001	<0.001
	East	-8.61 (-17.37 to 0.15)	0.054	
	North	-18.05 (-24.18 to -11.92)	<0.001	
	Outer island	-33.99 (-42.75 to -25.23)	<0.001	
	South	3.59 (-3.04 to 10.21)	0.289	
Per capita gross domestic product (GDP)	Intercept	-146.60 (-226.56 to -66.64)	<0.001	<0.001
	New Taiwan Dollar	2.31E-04 (1.32E-04 to 3.31E-04)	<0.001	
Population	Intercept	28.57 (25.28 to 31.86)	<0.001	<0.001
	Number of people	1.02E-05 (8.15E-06 to 1.23E-05)	<0.001	
Higher education	Intercept	37.72 (28.26 to 47.19)	<0.001	0.615
	%	0.06 (-0.16 to 0.28)	0.615	
Plain area of a county	Intercept	23.68 (20.29 to 27.07)	<0.001	<0.001

	10,000 m^2	3.62E-04 (3.06E-04 to 4.19E-04)	<0.001	
Population density over the plain area	Intercept	42.69 (39.90 to 45.49)	<0.001	<0.001
	Person / 10,000 m^2	-4.25E-04 (-6.54E-04 to -1.96E-04)	<0.001	
Population density over the county area	Intercept	42.60 (39.62 to 45.58)	<0.001	0.004
	Person / 1 km^2	-1.60E-03 (-2.70E-03 to -5.01E-04)	0.004	
Fertility rate	Intercept	60.41 (45.30 to 75.52)	<0.001	0.008
	‰	-0.70 (-1.20 to -0.19)	0.008	
Stray dogs	Intercept	21.01 (17.27 to 24.74)	<0.001	<0.001
	Number of stray dogs	2.73E-03 (2.30E-03 to 3.16E-03)	<0.001	
Total pets	Intercept	27.61 (24.03 to 31.18)	<0.001	<0.001
	Number of pet dogs and pet cats	1.14E-04 (8.89E-05 to 1.39E-04)	<0.001	
Total managers	Intercept	35.47 (32.44 to 38.49)	<0.001	<0.001
	Number of managers	0.35 (0.21 to 0.49)	<0.001	
Total ACOs	Intercept	27.02 (22.99 to 31.06)	<0.001	<0.001
	Number of animal control officers	2.25 (1.68 to 2.81)	<0.001	
Total vets	Intercept	26.68 (23.61 to 29.75)	<0.001	<0.001
	Number of veterinarians	2.57 (2.17 to 2.98)	<0.001	
Working day	Intercept	4.14 (-20.07 to 28.36)	0.738	0.004
	Number of working days monthly	1.72 (0.57 to 2.88)	0.004	

[1]: confidence interval

[2]: administrative divisions in Taiwan (i.e., metropolis, city, county, and outer island)

[3]: geographical divisions (i.e., north, south, west, east, and outer island)

Table S5 Table 6. Univariable linear regression results for the shelter animal outcomes per month over the maximum shelter capacity from 2018 to 2020.

**Covariate**	**Category**	**Estimate (95% CI[1])**	***P*-value**	**P-value for the covariate**
Year	Intercept	31.37 (27.51 to 35.23)	<0.001	<0.001
	Year	8.01 (5.03 to 11.00)	<0.001	
Month	Intercept	33.08 (27.80 to 38.35)	<0.001	0.008
	Month	0.97 (0.25 to 1.69)	0.008	
County	Intercept (Taipei)	36.97 (29.51 to 44.42)	<0.001	<0.001
	Chiayi County	16.24 (5.70 to 26.79)	0.003	
	Chiayi City	-15.60 (-26.14 to -5.05)	0.004	
	Changhua County	11.89 (1.35 to 22.44)	0.027	
	Hsinchu County	-5.46 (-16.01 to 5.08)	0.310	
	Hsinchu City	-25.98 (-36.52 to -15.43)	<0.001	
	Hualien County	7.03 (-3.51 to 17.57)	0.192	
	Kaohsiung	2.96 (-7.59 to 13.50)	0.583	
	Keelung County	-12.12 (-22.66 to -1.58)	0.025	
	Kinmen and Lienchiang County	-17.50 (-28.05 to -6.96)	0.001	
	Miaoli County	3.17 (-7.37 to 13.71)	0.556	
	Nantou County	-24.53 (-35.07 to -13.98)	<0.001	
	New Taipei	-11.90 (-22.44 to -1.36)	0.027	
	Penghu County	-27.96 (-38.50 to -17.41)	<0.001	
	Pingtung County	27.34 (16.80 to 37.89)	<0.001	

	Taichung	91.08 (80.53 to 101.62)	<0.001	
	Tainan	44.18 (33.63 to 54.72)	<0.001	
	Taitung County	1.27 (-9.27 to 11.81)	0.814	
	Taoyuan	8.80 (-1.75 to 19.34)	0.102	
	Yilan County	-0.29 (-10.83 to 10.25)	0.957	
	Yunlin County	-21.80 (-32.34 to -11.26)	<0.001	
Administrative division[2]	Intercept (County)	38.46 (35.22 to 41.69)	<0.001	<0.001
	City	-19.38 (-26.13 to -12.64)	<0.001	
	Metropolis	21.03 (15.74 to 26.32)	<0.001	
	Outer island	-24.22 (-32.15 to -16.29)	<0.001	
Geographical division[3]	Intercept (West)	48.93 (44.15 to 53.71)	<0.001	<0.001
	East	-7.81 (-16.76 to 1.14)	0.087	
	North	-18.67 (-24.93 to -12.41)	<0.001	
	Outer island	-34.69 (-43.64 to -25.75)	<0.001	
	South	3.06 (-3.70 to 9.83)	0.375	
Per capita gross domestic product (GDP)	Intercept	-174.07 (-255.37 to -92.76)	<0.001	<0.001
	New Taiwan Dollar	2.65E-04 (1.64E-04 to 3.65E-04)	<0.001	
Population	Intercept	28.07 (24.70 to 31.44)	<0.001	<0.001
	Number of people	1.01E-05 (7.93E-06 to 1.22E-05)	<0.001	
Higher education	Intercept	37.92 (28.26 to 47.58)	<0.001	0.757
	%	0.04 (-0.19 to 0.26)	0.757	
Plain area of a county	Intercept	23.27 (19.78 to 26.75)	<0.001	<0.001

	10,000 m^2	3.56E-04 (2.98E-04 to 4.15E-04)	<0.001	
Population density over the plain area	Intercept	41.89 (39.04 to 44.75)	<0.001	<0.001
	Person / 10,000 m^2	-4.06E-04 (-6.40E-04 to -1.72E-04)	<0.001	
Population density over the county area	Intercept	42.06 (39.02 to 45.11)	<0.001	0.003
	Person / 1 km^2	-1.69E-03 (-2.81E-03 to -5.70E-04)	0.003	
Fertility rate	Intercept	61.60 (46.19 to 77.01)	<0.001	0.004
	‰	-0.76 (-1.28 to -0.24)	0.004	
Stray dogs	Intercept	20.51 (16.67 to 24.34)	<0.001	<0.001
	Number of stray dogs	2.70E-03 (2.26E-03 to 3.14E-03)	<0.001	
Total pets	Intercept	27.21 (23.54 to 30.87)	<0.001	<0.001
	Number of pet dogs and pet cats	1.11E-04 (8.57E-05 to 1.37E-04)	<0.001	
Total managers	Intercept	34.81 (31.72 to 37.90)	<0.001	<0.001
	Number of managers	0.35 (0.21 to 0.49)	<0.001	
Total ACOs	Intercept	26.07 (21.95 to 30.19)	<0.001	<0.001
	Number of animal control officers	2.29 (1.72 to 2.87)	<0.001	
Total vets	Intercept	26.27 (23.11 to 29.43)	<0.001	<0.001
	Number of veterinarians	2.52 (2.10 to 2.94)	<0.001	
Working day	Intercept	6.04 (-18.70 to 30.77)	0.633	0.008
	Number of working days monthly	1.60 (0.42 to 2.78)	0.008	

[1]: confidence interval

[2]: administrative divisions in Taiwan (i.e., metropolis, city, county, and outer island)

[3]: geographical divisions (i.e., north, south, west, east, and outer island)

Table S5 Table 7. Univariable linear regression results for the number of adopted animals over the number of animals entering and staying at shelters per month from 2018 to 2020.

Covariate	Category	Estimate (95% CI[1])	*P*-value	P-value for the covariate
Year	Intercept	21.46 (19.96 to 22.96)	<0.001	<0.001
	Year	-2.64 (-3.79 to -1.48)	<0.001	
Month	Intercept	19.93 (17.89 to 21.97)	<0.001	0.229
	Month	-0.17 (-0.45 to 0.11)	0.229	
County	Intercept (Taipei)	13.35 (10.82 to 15.88)	<0.001	<0.001
	Chiayi County	-8.63 (-12.21 to -5.05)	<0.001	
	Chiayi City	10.77 (7.20 to 14.35)	<0.001	
	Changhua County	12.78 (9.20 to 16.35)	<0.001	
	Hsinchu County	10.28 (6.70 to 13.85)	<0.001	
	Hsinchu City	-1.54 (-5.12 to 2.04)	0.399	
	Hualien County	3.41 (-0.16 to 6.99)	0.062	
	Kaohsiung	8.48 (4.90 to 12.05)	<0.001	
	Keelung County	9.45 (5.87 to 13.02)	<0.001	
	Kinmen and Lienchiang County	-3.73 (-7.31 to -0.16)	0.041	
	Miaoli County	0.08 (-3.50 to 3.66)	0.965	
	Nantou County	-7.19 (-10.76 to -3.61)	<0.001	
	New Taipei	8.09 (4.52 to 11.67)	<0.001	
	Penghu County	-7.15 (-10.73 to -3.58)	<0.001	
	Pingtung County	13.63 (10.05 to 17.20)	<0.001	

	Taichung	13.04 (9.47 to 16.62)	<0.001	
	Tainan	8.07 (4.50 to 11.65)	<0.001	
	Taitung County	43.39 (39.82 to 46.97)	<0.001	
	Taoyuan	5.47 (1.89 to 9.05)	0.003	
	Yilan County	2.30 (-1.28 to 5.87)	0.209	
	Yunlin County	-6.04 (-9.61 to -2.46)	<0.001	
Administrative division[2]	Intercept (County)	19.75 (18.41 to 21.09)	<0.001	<0.001
	City	-0.17 (-2.97 to 2.62)	0.903	
	Metropolis	0.79 (-1.40 to 2.98)	0.479	
	Outer island	-11.84 (-15.13 to -8.56)	<0.001	
Geographical division[3]	Intercept (West)	15.88 (14.18 to 17.59)	<0.001	<0.001
	East	20.87 (17.67 to 24.07)	<0.001	
	North	2.33 (0.09 to 4.57)	0.042	
	Outer island	-7.98 (-11.18 to -4.78)	<0.001	
	South	3.93 (1.51 to 6.35)	0.001	
Per capita gross domestic product (GDP)	Intercept	89.78 (58.28 to 121.28)	<0.001	<0.001
	New Taiwan Dollar	-8.80E-05 (-1.27E-04 to -4.89E-05)	<0.001	
Population	Intercept	17.58 (16.22 to 18.95)	<0.001	0.013
	Number of people	1.11E-06 (2.37E-07 to 1.97E-06)	0.013	
Higher education	Intercept	21.85 (18.14 to 25.57)	<0.001	0.099
	%	-0.07 (-0.16 to 0.01)	0.099	
Plain area of a county	Intercept	18.54 (17.07 to 20.00)	<0.001	0.614

	10,000 m^2	6.31E-06 (-1.82E-05 to 3.08E-05)	0.614	
Population density over the plain area	Intercept	18.56 (17.45 to 19.67)	<0.001	0.359
	Person / 10,000 m^2	4.25E-05 (-4.82E-05 to 1.33E-04)	0.359	
Population density over the county area	Intercept	19.53 (18.35 to 20.70)	<0.001	0.045
	Person / 1 km^2	-4.45E-04 (-8.79E-04 to -1.11E-05)	0.045	
Fertility rate	Intercept	20.38 (14.41 to 26.36)	<0.001	0.605
	‰	-0.05 (-0.25 to 0.15)	0.605	
Stray dogs	Intercept	13.58 (12.03 to 15.12)	<0.001	<0.001
	Number of stray dogs	7.51E-04 (5.73E-04 to 9.29E-04)	<0.001	
Total pets	Intercept	17.25 (15.77 to 18.72)	<0.001	0.006
	Number of pet dogs and pet cats	1.44E-05 (4.16E-06 to 2.46E-05)	0.006	
Total managers	Intercept	18.60 (17.39 to 19.81)	<0.001	0.556
	Number of managers	0.02 (-0.04 to 0.07)	0.556	
Total ACOs	Intercept	20.77 (19.13 to 22.41)	<0.001	0.004
	Number of animal control officers	-0.34 (-0.57 to -0.10)	0.004	
Total vets	Intercept	18.12 (16.80 to 19.45)	<0.001	0.133
	Number of veterinarians	0.13 (-0.04 to 0.31)	0.133	
Working day	Intercept	13.19 (3.62 to 22.77)	0.007	0.247
	Number of working days monthly	0.27 (-0.19 to 0.73)	0.247	

[1]: confidence interval

[2]: administrative divisions in Taiwan (i.e., metropolis, city, county, and outer island)

[3]: geographical divisions (i.e., north, south, west, east, and outer island)

Table S5 Table 8. Univariable linear regression results for the indicator for the monthly workload of shelter veterinarians from 2018 to 2020.

Covariate	Category	Estimate (95% CI[1])	*P*-value	P-value for the covariate
Year	Intercept	62.10 (56.20 to 67.99)	<0.001	<0.001
	Year	12.01 (7.45 to 16.58)	<0.001	
Month	Intercept	62.42 (54.38 to 70.45)	<0.001	0.001
	Month	1.80 (0.71 to 2.89)	0.001	
County	Intercept (Taipei)	44.35 (32.24 to 56.45)	<0.001	<0.001
	Chiayi County	-14.04 (-31.16 to 3.08)	0.108	
	Chiayi City	7.35 (-9.77 to 24.46)	0.401	
	Changhua County	79.10 (61.98 to 96.21)	<0.001	
	Hsinchu County	119.93 (102.81 to 137.05)	<0.001	
	Hsinchu City	6.39 (-10.73 to 23.51)	0.465	
	Hualien County	85.12 (68.00 to 102.24)	<0.001	
	Kaohsiung	23.87 (6.75 to 40.99)	0.006	
	Keelung County	4.56 (-12.56 to 21.68)	0.602	
	Kinmen and Lienchiang County	2.26 (-14.86 to 19.38)	0.796	
	Miaoli County	77.33 (60.21 to 94.45)	<0.001	
	Nantou County	-9.26 (-26.38 to 7.86)	0.290	
	New Taipei	25.95 (8.83 to 43.07)	0.003	
	Penghu County	8.34 (-8.78 to 25.46)	0.340	
	Pingtung County	75.73 (58.62 to 92.85)	<0.001	
	Taichung	50.31 (33.19 to 67.43)	<0.001	

	Tainan	20.16 (3.05 to 37.28)	0.021	
	Taitung County	-2.38 (-19.50 to 14.74)	0.785	
	Taoyuan	67.65 (50.54 to 84.77)	<0.001	
	Yilan County	21.00 (3.89 to 38.12)	0.016	
	Yunlin County	-24.37 (-41.49 to -7.26)	0.005	
Administrative division[2]	Intercept (County)	85.17 (79.86 to 90.48)	<0.001	<0.001
	City	-34.72 (-45.77 to -23.66)	<0.001	
	Metropolis	-9.49 (-18.16 to -0.82)	0.032	
	Outer island	-35.52 (-48.52 to -22.51)	<0.001	
Geographical division[3]	Intercept (West)	78.97 (71.32 to 86.62)	<0.001	<0.001
	East	6.75 (-7.57 to 21.07)	0.356	
	North	0.45 (-9.57 to 10.47)	0.930	
	Outer island	-29.32 (-43.64 to -15.00)	<0.001	
	South	-12.01 (-22.83 to -1.18)	0.030	

[1]: confidence interval

[2]: administrative divisions in Taiwan (i.e., metropolis, city, county, and outer island)

[3]: geographical divisions (i.e., north, south, west, east, and outer island)
