# Supplementary material for: Trends in Animal Shelter Management, Adoption, and Animal Death in Taiwan from 2012 to 2020
Source: Animals (Basel). 2023 Apr 24;13(9):1451. doi: 10.3390/ani13091451 (PMC10177604; doi:10.3390/ani13091451)
Supplement: Supplementary file 1 [file animals-13-01451-s001.zip › Table S6.pdf]

Table S6. Final multivariable linear regression models.

Table S6 Table 1. Final multivariable linear regression model for logarithmic transformation of monthly shelter animal intakes over the maximum shelter capacity from 2012 to 2020.

Covariate	Category	Estimate (95% CI[1])	*P*-value
Intercept		4.20 (3.81 to 4.58)	<0.001
Year	Year	-0.08 (-0.12 to -0.05)	<0.001
County	Chiayi County	-0.23 (-0.67 to 0.21)	0.314
	Chiayi City	-0.40 (-0.84 to 0.04)	0.077
	Changhua County	-0.08 (-0.52 to 0.36)	0.715
	Hsinchu County	-0.19 (-0.62 to 0.25)	0.408
	Hsinchu City	-1.43 (-1.87 to -0.99)	<0.001
	Hualien County	0.11 (-0.33 to 0.55)	0.618
	Kaohsiung	0.07 (-0.37 to 0.50)	0.771
	Keelung County	-0.34 (-0.77 to 0.10)	0.137
	Kinmen and Lienchiang County	-0.72 (-1.16 to -0.28)	0.002
	Miaoli County	-0.02 (-0.45 to 0.42)	0.944
	Nantou County	-1.20 (-1.64 to -0.76)	<0.001
	New Taipei	-0.38 (-0.82 to 0.06)	0.090
	Penghu County	-1.34 (-1.78 to -0.90)	<0.001
	Pingtung County	0.61 (0.17 to 1.05)	0.007
	Taichung	1.12 (0.68 to 1.55)	<0.001
	Tainan	0.71 (0.27 to 1.15)	0.002
	Taitung County	0.17 (-0.27 to 0.61)	0.449
	Taoyuan	0.07 (-0.36 to 0.51)	0.739
	Yilan County	-0.02 (-0.45 to 0.42)	0.945
	Yunlin County	-0.97 (-1.41 to -0.53)	<0.001
Euthanasia	Yes/No	0.19 (-0.26 to 0.63)	0.412
County: Euthanasia	Chiayi County: Euthanasia	1.59 (1.00 to 2.18)	<0.001
	Chiayi City: Euthanasia	0.45 (-0.13 to 1.04)	0.132
	Changhua County: Euthanasia	0.85 (0.26 to 1.44)	0.005
	Hsinchu County: Euthanasia	1.04 (0.45 to 1.63)	<0.001
	Hsinchu City: Euthanasia	0.49 (-0.10 to 1.08)	0.107
	Hualien County: Euthanasia	0.84 (0.26 to 1.43)	0.006
	Kaohsiung: Euthanasia	-0.13 (-0.72 to 0.46)	0.657
	Keelung County: Euthanasia	0.61 (0.02 to 1.20)	0.044

	Kinmen and Lienchiang County: Euthanasia	-0.18 (-0.77 to 0.41)	0.553
	Miaoli County: Euthanasia	0.07 (-0.52 to 0.66)	0.817
	Nantou County: Euthanasia	1.65 (1.07 to 2.24)	<0.001
	New Taipei: Euthanasia	0.05 (-0.54 to 0.64)	0.861
	Penghu County: Euthanasia	0.10 (-0.49 to 0.69)	0.732
	Pingtung County: Euthanasia	1.17 (0.58 to 1.76)	<0.001
	Taichung: Euthanasia	-0.51 (-1.10 to 0.08)	0.091
	Tainan: Euthanasia	-0.19 (-0.78 to 0.40)	0.523
	Taitung County: Euthanasia	0.61 (0.02 to 1.20)	0.045
	Taoyuan: Euthanasia	0.13 (-0.45 to 0.72)	0.654
	Yilan County: Euthanasia	0.24 (-0.35 to 0.83)	0.432
	Yunlin County: Euthanasia	1.34 (0.76 to 1.93)	<0.001

[1]: confidence interval

Table S6 Table 2. Final multivariable linear regression model for logarithmic transformation of monthly shelter animal outcomes over the maximum shelter capacity from 2012 to 2020.

**Covariate**	**Category**	**Estimate (95% CI[1])**	***P*-value**
Intercept		4.13 (3.82 to 4.44)	<0.001
Year	Year	-0.09 (-0.11 to -0.06)	<0.001
County	Chiayi County	-0.67 (-1.03 to -0.32)	<0.001
	Chiayi City	-0.37 (-0.73 to -0.01)	0.043
	Changhua County	-0.13 (-0.48 to 0.23)	0.489
	Hsinchu County	-0.23 (-0.58 to 0.13)	0.213
	Hsinchu City	-1.61 (-1.97 to -1.26)	<0.001
	Hualien County	-0.26 (-0.61 to 0.10)	0.161
	Kaohsiung	0.03 (-0.32 to 0.39)	0.856
	Keelung County	-0.26 (-0.62 to 0.10)	0.153
	Kinmen and Lienchiang County	-1.59 (-1.95 to -1.24)	<0.001
	Miaoli County	-0.57 (-0.93 to -0.22)	0.002
	Nantou County	-1.41 (-1.77 to -1.06)	<0.001
	New Taipei	-0.31 (-0.66 to 0.05)	0.095
	Penghu County	-1.45 (-1.80 to -1.09)	<0.001
	Pingtung County	0.26 (-0.10 to 0.61)	0.162
	Taichung	0.73 (0.38 to 1.09)	<0.001
	Tainan	0.37 (0.01 to 0.73)	0.043
	Taitung County	0.25 (-0.11 to 0.60)	0.175
	Taoyuan	-0.31 (-0.66 to 0.05)	0.092
	Yilan County	-0.34 (-0.69 to 0.02)	0.066
	Yunlin County	-1.03 (-1.38 to -0.67)	<0.001
Euthanasia	Yes/No	0.11 (-0.25 to 0.47)	0.548
County: Euthanasia	Chiayi County: Euthanasia	2.06 (1.58 to 2.54)	<0.001
	Chiayi City: Euthanasia	0.52 (0.04 to 0.99)	0.036
	Changhua County: Euthanasia	0.86 (0.39 to 1.34)	<0.001
	Hsinchu County: Euthanasia	1.21 (0.73 to 1.69)	<0.001
	Hsinchu City: Euthanasia	0.74 (0.27 to 1.22)	0.003
	Hualien County: Euthanasia	1.25 (0.77 to 1.72)	<0.001
	Kaohsiung: Euthanasia	-0.28 (-0.76 to 0.19)	0.246
	Keelung County: Euthanasia	0.57 (0.09 to 1.05)	0.021
	Kinmen and Lienchiang County: Euthanasia	0.48 (4.63E-03 to 0.96)	0.050

	Miaoli County: Euthanasia	0.68 (0.20 to 1.16)	0.006
	Nantou County: Euthanasia	1.93 (1.45 to 2.41)	<0.001
	New Taipei: Euthanasia	-0.03 (-0.51 to 0.44)	0.889
	Penghu County: Euthanasia	-0.02 (-0.50 to 0.45)	0.922
	Pingtung County: Euthanasia	1.68 (1.20 to 2.16)	<0.001
	Taichung: Euthanasia	-0.05 (-0.53 to 0.43)	0.834
	Tainan: Euthanasia	0.25 (-0.23 to 0.73)	0.305
	Taitung County: Euthanasia	0.61 (0.13 to 1.09)	0.014
	Taoyuan: Euthanasia	0.63 (0.15 to 1.11)	0.011
	Yilan County: Euthanasia	0.54 (0.06 to 1.01)	0.030
	Yunlin County: Euthanasia	1.54 (1.06 to 2.01)	<0.001

[1]: confidence interval

Table S6 Table 3. Final multivariable linear regression model for the number of adopted animals over the shelter animal intakes per month from 2012 to 2020.

**Covariate**	**Category**	**Estimate (95% CI[1])**	***P*-value**
Intercept		18.20 (-20.95 to 57.35)	0.364
Year	Year	5.11 (3.79 to 6.43)	<0.001
County	Chiayi County	-12.60 (-33.48 to 8.27)	0.238
	Chiayi City	14.22 (-5.67 to 34.11)	0.163
	Changhua County	-32.30 (-50.05 to -14.55)	<0.001
	Hsinchu County	-12.39 (-30.09 to 5.31)	0.172
	Hsinchu City	-21.01 (-38.82 to -3.20)	0.022
	Hualien County	-20.37 (-38.81 to -1.93)	0.032
	Kaohsiung	-5.23 (-24.25 to 13.80)	0.591
	Keelung County	-2.89 (-24.25 to 18.47)	0.791
	Kinmen and Lienchiang County	-58.33 (-76.07 to -40.59)	<0.001
	Miaoli County	-35.85 (-53.92 to -17.77)	<0.001
	Nantou County	-29.77 (-49.22 to -10.31)	0.003
	New Taipei	5.11 (-13.36 to 23.58)	0.589
	Penghu County	-36.60 (-54.29 to -18.91)	<0.001
	Pingtung County	-27.74 (-48.77 to -6.71)	0.011
	Taichung	-19.58 (-37.84 to -1.33)	0.037
	Tainan	-4.47 (-23.61 to 14.67)	0.648
	Taitung County	6.52 (-12.40 to 25.44)	0.500
	Taoyuan	-14.18 (-31.88 to 3.53)	0.118
	Yilan County	-30.97 (-50.04 to -11.91)	0.002
	Yunlin County	-6.46 (-25.64 to 12.72)	0.510
Fertility rate	‰	1.10 (0.21 to 2.00)	0.016

[1]: confidence interval

Table S6 Table 4. Final multivariable linear regression model for square root transformation of the number of unassisted death animals over the shelter animal intakes per year from 2012 to 2020.

**Covariate**	**Category**	**Estimate (95% CI[1])**	***P*-value**
Intercept		3.80 (2.81 to 4.79)	<0.001
Year	Year	-0.06 (-0.34 to 0.21)	0.659
County	Chiayi County	2.21 (0.81 to 3.62)	0.003
	Chiayi City	-0.61 (-2.01 to 0.79)	0.398
	Changhua County	1.65 (0.25 to 3.05)	0.023
	Hsinchu County	-0.61 (-2.01 to 0.79)	0.397
	Hsinchu City	0.62 (-0.78 to 2.02)	0.387
	Hualien County	0.15 (-1.25 to 1.55)	0.834
	Kaohsiung	2.51 (1.11 to 3.91)	<0.001
	Keelung County	0.18 (-1.22 to 1.58)	0.801
	Kinmen and Lienchiang County	0.25 (-1.15 to 1.65)	0.729
	Miaoli County	2.21 (0.81 to 3.61)	0.003
	Nantou County	2.55 (1.15 to 3.96)	<0.001
	New Taipei	0.94 (-0.46 to 2.34)	0.191
	Penghu County	1.30 (-0.10 to 2.71)	0.071
	Pingtung County	-1.75 (-3.15 to -0.35)	0.016
	Taichung	-0.30 (-1.70 to 1.10)	0.679
	Tainan	0.31 (-1.09 to 1.71)	0.667
	Taitung County	-0.44 (-1.84 to 0.96)	0.542
	Taoyuan	-0.52 (-1.92 to 0.88)	0.469
	Yilan County	0.76 (-0.64 to 2.16)	0.289
	Yunlin County	-3.47 (-4.87 to -2.07)	<0.001
Year: County	Year: Chiayi County	-0.27 (-0.66 to 0.12)	0.173
	Year: Chiayi City	0.03 (-0.36 to 0.42)	0.872
	Year: Changhua County	-0.24 (-0.62 to 0.15)	0.238
	Year: Hsinchu County	0.20 (-0.19 to 0.59)	0.322
	Year: Hsinchu City	0.04 (-0.35 to 0.43)	0.854
	Year: Hualien County	-0.17 (-0.56 to 0.22)	0.396
	Year: Kaohsiung	-0.56 (-0.95 to -0.17)	0.006
	Year: Keelung County	0.13 (-0.26 to 0.51)	0.529
	Year: Kinmen and Lienchiang County	-0.20 (-0.59 to 0.19)	0.317
	Year: Miaoli County	-0.20 (-0.59 to 0.19)	0.310

	Year: Nantou County	-0.51 (-0.90 to -0.12)	0.011
	Year: New Taipei	-0.28 (-0.66 to 0.11)	0.168
	Year: Penghu County	0.27 (-0.12 to 0.66)	0.175
	Year: Pingtung County	0.15 (-0.24 to 0.54)	0.456
	Year: Taichung	0.09 (-0.30 to 0.48)	0.640
	Year: Tainan	-0.12 (-0.50 to 0.27)	0.562
	Year: Taitung County	-0.23 (-0.62 to 0.16)	0.250
	Year: Taoyuan	0.01 (-0.38 to 0.40)	0.960
	Year: Yilan County	-0.05 (-0.44 to 0.34)	0.805
	Year: Yunlin County	1.16 (0.77 to 1.55)	<0.001

[1]: confidence interval

Table S6 Table 5. Final multivariable linear regression model for logarithmic transformation of monthly shelter animal intakes over the maximum shelter capacity from 2018 to 2020.

**Covariate**	**Category**	**Estimate (95% CI[1])**	***P*-value**
Intercept		2.70 (2.33 to 3.06)	<0.001
Year	Year	0.04 (-0.53 to 0.62)	0.882
County	Chiayi County	-0.87 (-1.19 to -0.55)	<0.001
	Chiayi City	-0.78 (-1.10 to -0.46)	<0.001
	Changhua County	-0.92 (-1.24 to -0.60)	<0.001
	Hsinchu County	-0.55 (-0.87 to -0.23)	<0.001
	Hsinchu City	-1.91 (-2.23 to -1.59)	<0.001
	Hualien County	-0.25 (-0.57 to 0.07)	0.126
	Kaohsiung	-0.15 (-0.47 to 0.17)	0.359
	Keelung County	-0.38 (-0.70 to -0.05)	0.022
	Kinmen and Lienchiang County	-0.85 (-1.17 to -0.53)	<0.001
	Miaoli County	-0.46 (-0.78 to -0.14)	0.005
	Nantou County	-1.70 (-2.02 to -1.38)	<0.001
	New Taipei	-0.46 (-0.78 to -0.14)	0.005
	Penghu County	-1.45 (-1.78 to -1.13)	<0.001
	Pingtung County	-1.16 (-1.48 to -0.84)	<0.001
	Taichung	1.01 (0.69 to 1.33)	<0.001
	Tainan	0.65 (0.33 to 0.97)	<0.001
	Taitung County	-0.08 (-0.40 to 0.24)	0.620
	Taoyuan	-0.16 (-0.48 to 0.16)	0.321
	Yilan County	-0.36 (-0.68 to -0.04)	0.027
	Yunlin County	-0.72 (-1.05 to -0.40)	<0.001
Working Day	Number of working days monthly	0.05 (0.04 to 0.06)	<0.001
$Year^2$	$Year^2$	-0.07 (-0.34 to 0.21)	0.635
Year: County	Year: Chiayi County	-0.89 (-1.71 to -0.07)	0.033
	Year: Chiayi City	0.09 (-0.73 to 0.91)	0.828
	Year: Changhua County	1.86 (1.05 to 2.68)	<0.001
	Year: Hsinchu County	0.18 (-0.64 to 0.99)	0.674
	Year: Hsinchu City	0.82 (4.45E-03 to 1.64)	0.049
	Year: Hualien County	0.26 (-0.56 to 1.07)	0.539
	Year: Kaohsiung	0.36 (-0.45 to 1.18)	0.382
	Year: Keelung County	-0.33 (-1.14 to 0.49)	0.435

	Year: Kinmen and Lienchiang County	-0.10 (-0.92 to 0.72)	0.812
	Year: Miaoli County	0.18 (-0.64 to 1.00)	0.669
	Year: Nantou County	-4.43E-03 (-0.82 to 0.81)	0.992
	Year: New Taipei	0.18 (-0.64 to 1.00)	0.669
	Year: Penghu County	0.28 (-0.53 to 1.10)	0.495
	Year: Pingtung County	2.56 (1.74 to 3.38)	<0.001
	Year: Taichung	0.28 (-0.54 to 1.10)	0.499
	Year: Tainan	0.21 (-0.60 to 1.03)	0.607
	Year: Taitung County	-0.16 (-0.98 to 0.66)	0.702
	Year: Taoyuan	0.43 (-0.39 to 1.25)	0.302
	Year: Yilan County	0.27 (-0.54 to 1.09)	0.512
	Year: Yunlin County	0.52 (-0.30 to 1.34)	0.211
$Year^2$: County	$Year^2$: Chiayi County	0.88 (0.49 to 1.27)	<0.001
	$Year^2$: Chiayi City	0.03 (-0.36 to 0.43)	0.862
	$Year^2$: Changhua County	-0.58 (-0.97 to -0.18)	0.004
	$Year^2$: Hsinchu County	0.09 (-0.30 to 0.48)	0.656
	$Year^2$: Hsinchu City	-0.13 (-0.52 to 0.26)	0.515
	$Year^2$: Hualien County	0.02 (-0.38 to 0.41)	0.931
	$Year^2$: Kaohsiung	-0.10 (-0.49 to 0.29)	0.612
	$Year^2$: Keelung County	0.13 (-0.27 to 0.52)	0.531
	$Year^2$: Kinmen and Lienchiang County	0.16 (-0.23 to 0.56)	0.413
	$Year^2$: Miaoli County	0.15 (-0.24 to 0.54)	0.460
	$Year^2$: Nantou County	0.27 (-0.12 to 0.66)	0.175
	$Year^2$: New Taipei	-0.09 (-0.48 to 0.31)	0.669
	$Year^2$: Penghu County	-0.05 (-0.44 to 0.35)	0.815
	$Year^2$: Pingtung County	-0.78 (-1.17 to -0.39)	<0.001
	$Year^2$: Taichung	-0.08 (-0.47 to 0.32)	0.705
	$Year^2$: Tainan	-0.07 (-0.47 to 0.32)	0.716
	$Year^2$: Taitung County	0.12 (-0.28 to 0.51)	0.558
	$Year^2$: Taoyuan	-0.06 (-0.46 to 0.33)	0.748
	$Year^2$: Yilan County	0.02 (-0.37 to 0.41)	0.925
	$Year^2$: Yunlin County	-0.55 (-0.94 to -0.16)	0.006

[1]: confidence interval

Table S6 Table 6. Final multivariable linear regression model for logarithmic transformation of monthly shelter animal outcomes over the maximum shelter capacity from 2018 to 2020.

Covariate	Category	Estimate (95% CI[1])	*P*-value
Intercept		2.66 (2.28 to 3.04)	<0.001
Year	Year	-0.06 (-0.67 to 0.54)	0.834
County	Chiayi County	-0.90 (-1.23 to -0.56)	<0.001
	Chiayi City	-0.67 (-1.01 to -0.33)	<0.001
	Changhua County	-0.86 (-1.20 to -0.53)	<0.001
	Hsinchu County	-0.47 (-0.81 to -0.14)	0.006
	Hsinchu City	-2.01 (-2.35 to -1.67)	<0.001
	Hualien County	-0.19 (-0.52 to 0.15)	0.271
	Kaohsiung	-0.15 (-0.49 to 0.19)	0.380
	Keelung County	-0.33 (-0.67 to 2.79E-03)	0.052
	Kinmen and Lienchiang County	-0.82 (-1.16 to -0.48)	<0.001
	Miaoli County	-0.34 (-0.67 to 7.84E-04)	0.051
	Nantou County	-1.71 (-2.05 to -1.37)	<0.001
	New Taipei	-0.40 (-0.73 to -0.06)	0.021
	Penghu County	-1.65 (-1.99 to -1.32)	<0.001
	Pingtung County	-1.00 (-1.33 to -0.66)	<0.001
	Taichung	1.06 (0.73 to 1.40)	<0.001
	Tainan	0.63 (0.29 to 0.97)	<0.001
	Taitung County	-0.03 (-0.37 to 0.30)	0.852
	Taoyuan	-0.13 (-0.47 to 0.20)	0.433
	Yilan County	-0.51 (-0.85 to -0.18)	0.003
	Yunlin County	-0.93 (-1.27 to -0.60)	<0.001
Working Day	Number of working days monthly	0.05 (0.03 to 0.06)	<0.001
$Year^2$	$Year^2$	-3.04E-03 (-0.29 to 0.29)	0.984
Year: County	Year: Chiayi County	-1.00 (-1.86 to -0.15)	0.022
	Year: Chiayi City	-0.18 (-1.04 to 0.67)	0.675
	Year: Changhua County	1.63 (0.77 to 2.49)	<0.001
	Year: Hsinchu County	0.12 (-0.74 to 0.97)	0.787
	Year: Hsinchu City	0.70 (-0.15 to 1.56)	0.108
	Year: Hualien County	0.47 (-0.38 to 1.33)	0.280
	Year: Kaohsiung	0.46 (-0.40 to 1.32)	0.293
	Year: Keelung County	-0.20 (-1.05 to 0.66)	0.651

	Year: Kinmen and Lienchiang County	-0.37 (-1.23 to 0.49)	0.396
	Year: Miaoli County	-0.09 (-0.95 to 0.77)	0.836
	Year: Nantou County	0.19 (-0.67 to 1.05)	0.664
	Year: New Taipei	0.20 (-0.66 to 1.05)	0.652
	Year: Penghu County	0.51 (-0.34 to 1.37)	0.241
	Year: Pingtung County	2.53 (1.67 to 3.39)	<0.001
	Year: Taichung	0.41 (-0.45 to 1.26)	0.354
	Year: Tainan	0.42 (-0.43 to 1.28)	0.332
	Year: Taitung County	-0.06 (-0.92 to 0.80)	0.889
	Year: Taoyuan	0.55 (-0.31 to 1.41)	0.208
	Year: Yilan County	0.67 (-0.19 to 1.52)	0.128
	Year: Yunlin County	1.00 (0.15 to 1.86)	0.022
$Year^2$: County	$Year^2$: Chiayi County	0.93 (0.51 to 1.34)	<0.001
	$Year^2$: Chiayi City	0.16 (-0.25 to 0.58)	0.436
	$Year^2$: Changhua County	-0.44 (-0.85 to -0.03)	0.037
	$Year^2$: Hsinchu County	0.08 (-0.33 to 0.49)	0.699
	$Year^2$: Hsinchu City	-0.04 (-0.45 to 0.37)	0.842
	$Year^2$: Hualien County	-0.10 (-0.51 to 0.31)	0.626
	$Year^2$: Kaohsiung	-0.16 (-0.57 to 0.26)	0.460
	$Year^2$: Keelung County	0.05 (-0.36 to 0.46)	0.801
	$Year^2$: Kinmen and Lienchiang County	0.27 (-0.14 to 0.69)	0.193
	$Year^2$: Miaoli County	0.25 (-0.16 to 0.66)	0.235
	$Year^2$: Nantou County	0.17 (-0.24 to 0.58)	0.410
	$Year^2$: New Taipei	-0.11 (-0.52 to 0.30)	0.603
	$Year^2$: Penghu County	-0.23 (-0.64 to 0.18)	0.273
	$Year^2$: Pingtung County	-0.79 (-1.20 to -0.37)	<0.001
	$Year^2$: Taichung	-0.14 (-0.55 to 0.27)	0.509
	$Year^2$: Tainan	-0.16 (-0.57 to 0.25)	0.438
	$Year^2$: Taitung County	0.06 (-0.35 to 0.47)	0.772
	$Year^2$: Taoyuan	-0.13 (-0.54 to 0.28)	0.535
	$Year^2$: Yilan County	-0.14 (-0.55 to 0.28)	0.519
	$Year^2$: Yunlin County	-0.76 (-1.17 to -0.35)	<0.001

[1]: confidence interval

Table S6 Table 7. Final multivariable linear regression model for square root transformation of the number of adopted animals over the number of animals entering and staying at shelters per month from 2018 to 2020.

**Covariate**	**Category**	**Estimate (95% CI[1])**	***P*-value**
Intercept		3.47 (2.83 to 4.11)	<0.001
Year	Year	-0.76 (-1.77 to 0.26)	0.144
County	Chiayi County	-1.65 (-2.21 to -1.09)	<0.001
	Chiayi City	1.02 (0.46 to 1.58)	<0.001
	Changhua County	0.54 (-0.02 to 1.10)	0.060
	Hsinchu County	1.77 (1.21 to 2.33)	<0.001
	Hsinchu City	-0.28 (-0.85 to 0.28)	0.324
	Hualien County	-0.09 (-0.65 to 0.48)	0.763
	Kaohsiung	0.80 (0.24 to 1.36)	0.005
	Keelung County	0.52 (-0.05 to 1.08)	0.073
	Kinmen and Lienchiang County	-0.62 (-1.18 to -0.06)	0.031
	Miaoli County	-0.54 (-1.10 to 0.03)	0.062
	Nantou County	-1.62 (-2.18 to -1.06)	<0.001
	New Taipei	0.79 (0.23 to 1.35)	0.006
	Penghu County	-1.56 (-2.13 to -1.00)	<0.001
	Pingtung County	2.05 (1.49 to 2.62)	<0.001
	Taichung	0.77 (0.21 to 1.34)	0.007
	Tainan	1.30 (0.74 to 1.86)	<0.001
	Taitung County	3.45 (2.89 to 4.02)	<0.001
	Taoyuan	0.78 (0.22 to 1.34)	0.007
	Yilan County	-0.77 (-1.34 to -0.21)	0.007
	Yunlin County	-0.99 (-1.55 to -0.42)	<0.001
Working Day	Number of working days monthly	0.03 (0.01 to 0.06)	0.011
$Year^2$	$Year^2$	0.15 (-0.34 to 0.63)	0.557
Year: County	Year: Chiayi County	0.55 (-0.88 to 1.99)	0.451
	Year: Chiayi City	0.99 (-0.45 to 2.42)	0.177
	Year: Changhua County	1.28E-03 (-1.43 to 1.44)	0.999
	Year: Hsinchu County	-1.68 (-3.11 to -0.24)	0.022
	Year: Hsinchu City	-0.28 (-1.72 to 1.15)	0.700
	Year: Hualien County	1.25 (-0.19 to 2.68)	0.089
	Year: Kaohsiung	0.90 (-0.54 to 2.33)	0.222
	Year: Keelung County	1.99 (0.56 to 3.43)	0.007

	Year: Kinmen and Lienchiang County	-0.06 (-1.50 to 1.38)	0.935
	Year: Miaoli County	0.49 (-0.95 to 1.93)	0.504
	Year: Nantou County	0.52 (-0.92 to 1.95)	0.479
	Year: New Taipei	0.51 (-0.93 to 1.94)	0.488
	Year: Penghu County	1.73 (0.29 to 3.16)	0.018
	Year: Pingtung County	-2.23 (-3.66 to -0.79)	0.002
	Year: Taichung	2.19 (0.75 to 3.62)	0.003
	Year: Tainan	-0.35 (-1.79 to 1.09)	0.633
	Year: Taitung County	0.98 (-0.45 to 2.42)	0.181
	Year: Taoyuan	-0.51 (-1.94 to 0.93)	0.490
	Year: Yilan County	2.66 (1.22 to 4.09)	<0.001
	Year: Yunlin County	1.33 (-0.10 to 2.77)	0.070
$Year^2$: County	$Year^2$: Chiayi County	-0.27 (-0.96 to 0.42)	0.443
	$Year^2$: Chiayi City	-0.46 (-1.15 to 0.23)	0.189
	$Year^2$: Changhua County	0.52 (-0.17 to 1.21)	0.142
	$Year^2$: Hsinchu County	0.63 (-0.06 to 1.32)	0.075
	$Year^2$: Hsinchu City	0.20 (-0.49 to 0.89)	0.578
	$Year^2$: Hualien County	-0.43 (-1.12 to 0.26)	0.221
	$Year^2$: Kaohsiung	-0.40 (-1.09 to 0.29)	0.256
	$Year^2$: Keelung County	-0.85 (-1.54 to -0.16)	0.015
	$Year^2$: Kinmen and Lienchiang County	0.05 (-0.64 to 0.74)	0.889
	$Year^2$: Miaoli County	0.03 (-0.66 to 0.72)	0.942
	$Year^2$: Nantou County	-0.04 (-0.73 to 0.65)	0.918
	$Year^2$: New Taipei	-0.17 (-0.86 to 0.52)	0.626
	$Year^2$: Penghu County	-0.90 (-1.59 to -0.21)	0.011
	$Year^2$: Pingtung County	0.92 (0.23 to 1.61)	0.009
	$Year^2$: Taichung	-0.89 (-1.58 to -0.20)	0.011
	$Year^2$: Tainan	-3.53E-03 (-0.69 to 0.69)	0.992
	$Year^2$: Taitung County	-0.32 (-1.01 to 0.37)	0.365
	$Year^2$: Taoyuan	0.25 (-0.44 to 0.94)	0.479
	$Year^2$: Yilan County	-0.96 (-1.65 to -0.27)	0.007
	$Year^2$: Yunlin County	-0.89 (-1.58 to -0.20)	0.011

[1]: confidence interval

Supplementary File 5 Table 8. Final multivariable linear regression model for logarithmic transformation of monthly workload of shelter veterinarians from 2018 to 2020.

**Covariate**	**Category**	**Estimate (95% CI[1])**	***P*-value**
Intercept		3.79 (3.56 to 4.03)	<0.001
Year	Year	-0.13 (-0.29 to 0.03)	0.121
Month	February	-0.21 (-0.35 to -0.07)	0.004
	March	0.10 (-0.04 to 0.24)	0.175
	April	0.07 (-0.07 to 0.21)	0.336
	May	0.17 (0.03 to 0.31)	0.020
	June	0.10 (-0.04 to 0.24)	0.173
	July	0.09 (-0.05 to 0.24)	0.207
	August	0.13 (-0.01 to 0.27)	0.076
	September	0.20 (0.05 to 0.34)	0.008
	October	0.27 (0.13 to 0.41)	<0.001
	November	0.22 (0.07 to 0.36)	0.003
	December	0.19 (0.05 to 0.33)	0.010
County	Chiayi County	-1.32 (-1.62 to -1.02)	<0.001
	Chiayi City	-0.08 (-0.38 to 0.22)	0.615
	Changhua County	-0.01 (-0.31 to 0.28)	0.922
	Hsinchu County	0.87 (0.57 to 1.17)	<0.001
	Hsinchu City	-0.16 (-0.46 to 0.14)	0.298
	Hualien County	0.26 (-0.04 to 0.56)	0.085
	Kaohsiung	0.07 (-0.23 to 0.37)	0.655
	Keelung County	0.11 (-0.19 to 0.41)	0.476
	Kinmen and Lienchiang County	-0.02 (-0.32 to 0.28)	0.882
	Miaoli County	0.44 (0.14 to 0.74)	0.004
	Nantou County	-0.75 (-1.04 to -0.45)	<0.001
	New Taipei	0.63 (0.33 to 0.93)	<0.001
	Penghu County	0.37 (0.07 to 0.67)	0.015
	Pingtung County	-0.36 (-0.66 to -0.06)	0.018
	Taichung	0.62 (0.32 to 0.92)	<0.001
	Tainan	0.13 (-0.17 to 0.43)	0.381
	Taitung County	0.15 (-0.15 to 0.45)	0.321
	Taoyuan	0.72 (0.42 to 1.02)	<0.001
	Yilan County	0.03 (-0.27 to 0.33)	0.829
	Yunlin County	-1.08 (-1.38 to -0.78)	<0.001

Year: County	Year: Chiayi County	0.51 (0.28 to 0.74)	<0.001
	Year: Chiayi City	0.20 (-0.03 to 0.43)	0.093
	Year: Changhua County	0.80 (0.57 to 1.03)	<0.001
	Year: Hsinchu County	0.37 (0.14 to 0.61)	0.002
	Year: Hsinchu City	0.21 (-0.02 to 0.44)	0.074
	Year: Hualien County	0.68 (0.45 to 0.91)	<0.001
	Year: Kaohsiung	0.34 (0.11 to 0.57)	0.004
	Year: Keelung County	-0.04 (-0.27 to 0.19)	0.746
	Year: Kinmen and Lienchiang County	0.04 (-0.19 to 0.27)	0.735
	Year: Miaoli County	0.49 (0.26 to 0.72)	<0.001
	Year: Nantou County	0.41 (0.17 to 0.64)	<0.001
	Year: New Taipei	-0.20 (-0.43 to 0.04)	0.098
	Year: Penghu County	-0.26 (-0.49 to -0.03)	0.026
	Year: Pingtung County	1.01 (0.78 to 1.24)	<0.001
	Year: Taichung	0.14 (-0.09 to 0.37)	0.231
	Year: Tainan	0.24 (4.44E-03 to 0.47)	0.046
	Year: Taitung County	-0.30 (-0.54 to -0.07)	0.010
	Year: Taoyuan	0.21 (-0.03 to 0.44)	0.083
	Year: Yilan County	0.33 (0.09 to 0.56)	0.006
	Year: Yunlin County	0.13 (-0.10 to 0.36)	0.272

[1]: confidence interval
